# Predicting the Influences of Depression and Sexual Stigma on Motivation to Get Vaccinated against COVID-19 in Lesbian, Gay, and Bisexual Young Adults: A 4-Year Follow-Up Study

**DOI:** 10.3390/vaccines11091430

**Published:** 2023-08-28

**Authors:** Yen-Ju Lin, Yu-Ping Chang, Cheng-Fang Yen

**Affiliations:** 1 Department of Psychiatry, Kaohsiung Medical University Hospital, Kaohsiung 80708, Taiwan; 2 Department of Psychiatry, School of Medicine, Kaohsiung Medical University, Kaohsiung 80708, Taiwan; 3 School of Nursing, The State University of New York, University at Buffalo, New York, NY 14214-8013, USA; 4 College of Professional Studies, National Pingtung University of Science and Technology, Pingtung 91201, Taiwan

**Keywords:** lesbian, gay, bisexual, vaccine, depression, sexual stigma, COVID-19, psychological well-being

## Abstract

Vaccination is a crucial preventive measure against COVID-19. However, limited research has focused on identifying the factors predicting motivation to get vaccinated against COVID-19 (MoVAC-19) among lesbian, gay, and bisexual (LGB) individuals. This study examined the predictive effects of depression and sexual stigma (i.e., perceived sexual stigma from family members, perceived sexual orientation microaggression, and internalized sexual stigma) before the COVID-19 pandemic on MoVAC-19 among LGB individuals 4 years later during the COVID-19 pandemic in Taiwan. Baseline data related to depression and sexual stigma were collected in 2018 and 2019. Depression was assessed using the 20-item Mandarin Chinese version of the Center for Epidemiologic Studies Depression Scale. Perceived sexual stigma from family members was assessed using the Homosexuality-Related Stigma Scale. Internalized sexual stigma was assessed using the Measure of Internalized Sexual Stigma for Lesbians and Gay Men. Perceived sexual orientation microaggression was assessed using the Sexual Orientation Microaggression Inventory. Participant MoVAC-19 during the pandemic was assessed using the nine-item Motors of COVID-19 Vaccination Acceptance Scale. The associations of depression and sexual stigma at baseline with MoVAC-19 at follow-up were examined through multivariate linear regression analysis. Internalized sexual stigma was negatively associated with MoVAC-19, whereas perceived sexual orientation microaggression was positively associated with MoVAC-19. Depression and perceived sexual stigma from family members were not significantly associated with MoVAC-19. Although male sex and older age were positively associated with increased MoVAC-19, sex and age did not moderate the relationship between sexual stigma and motivation to get vaccinated. Among LGB individuals, sexual stigma experiences should be considered when developing intervention strategies aimed at enhancing MoVAC-19.

## 1. Introduction

COVID-19 has had a major impact on human health and daily life. According to the World Health Organization (WHO), as of 14 July 2023, more than 760 million people had contracted COVID-19, with approximately 7 million resultant deaths worldwide [1]. Although on 3 May 2023, the WHO declared that COVID-19 was no longer a public health emergency of international concern [2], more than 2.5 million new cases and approximately 15,000 deaths were reported during the period from 8 May to 9 July 2023 [3]. Vaccination against COVID-19 is a crucial measure for reducing the risk of infection and hospitalization [4]. However, COVID-19 vaccine hesitancy remains prevalent. A meta-analysis of 56 published articles revealed a global prevalence of COVID-19 vaccine hesitancy of 25% [5]. Therefore, assessing individual motivations and the factors influencing motivations to get vaccinated against COVID-19 (MoVAC-19) and developing programs to enhance MoVAC-19 are essential.

Several theories, such as the theory of planned behavior (TPB) [6,7] and the health belief model (HBM) [8], have been used to examine critical factors relevant to MoVAC-19. Regarding the TPB, a systematic review and meta-analysis of 43 articles examining the factors relevant to the intention to get vaccinated against COVID-19 revealed that attitudes toward COVID-19 vaccines exhibited the strongest correlation with vaccination intention, followed by subjective norms of vaccination against COVID-19 and subjectively behavioral control of vaccination [9]. Regarding the HBM, a systematic review and meta-analysis of 19 articles revealed that increased perceived susceptibility to COVID-19 infection, perceived severity of COVID-19, perceived benefits of vaccination against COVID-19, and vaccination cues positively predicted the intention to get vaccinated against COVID-19, whereas increased perceived barriers to vaccination negatively predicted the intention to get vaccinated against COVID-19 [10]. According to theory-driven studies, MoVAC-19 is influenced by individual, environmental, and individual–environmental interaction factors [8,9,10]. Consequently, according to ecological systems theory [11], marginal populations may exhibit unique factors relevant to their MoVAC-19.

Lesbian, gay, and bisexual (LGB) individuals represent a marginal population that has been strongly impacted by COVID-19 [12]. A study on LGB, transgender, queer, and other-sexual-identity individuals living in New York between 30 June and 13 December 2021 found that 19% of participants had not received any dose of a COVID-19 vaccine, [13]. The National Immunization Survey Adult COVID Module, United States between 29 August and 30 October 2021 demonstrated that 8.2% of gay or lesbian individuals and 10.2% of bisexual individuals reported that they probably or definitely would not get vaccinated [14]. In addition, a study in the United States between 13 May 2021 and 9 January 2022 demonstrated that sexual minority individuals had a lower intention of receiving the COVID-19 vaccine (65.62%) than heterosexual individuals (67.56%) [15], whereas a study in Taiwan between 15 October 2020 and 21 December 2020 found that LGB individuals had a higher level of intention to get vaccinated against COVID-19 compared with heterosexual individuals [16].

Multiple studies have identified several common factors associated with MoVAC-19 in both LGB individuals and the general population. These factors include male sex, older age, educational attainment beyond an undergraduate degree, being in a relationship, being employed, holding positive attitudes toward the safety of COVID-19 vaccines, a positive HIV status, a negative COVID-19 infection history, and having chronic physical illnesses [5,14,17,18,19]. The WHO also recommended the individuals with significant comorbidities (e.g., diabetes, heart disease, serious immunocompromising conditions, severe obesity, and pregnancy) as the high priority group for vaccination [20]. Other studies have also identified unique factors associated with MoVAC-19 among LGB individuals. For instance, a review study of 17 articles revealed a cross-sectional association of concerns regarding social stigma due to sexual minority status with reduced intention to get vaccinated against COVID-19 among LGB individuals [21]. Perceived sexual stigma may prevent LGB individuals from accessing appropriate vaccine knowledge and administration information [20]. Furthermore, in LGB individuals, mild depressive symptoms are cross-sectionally associated with MoVAC-19 [15]. Depressive symptoms may heighten concerns about contracting COVID-19 and thus may increase MoVAC-19.

Several problems related to MoVAC-19 in LGB individuals have been identified. First, few prospective studies have examined the predictors of MoVAC-19 in LGB individuals. In their prospective studies, Prestage et al. [22] and Zhang et al. [23] examined the predictive effects of multiple factors—namely demographic characteristics, self-protective behaviors against COVID-19, concerns over contracting COVID-9, social connectedness and sexual activities during the pandemic, attitudes toward vaccination and restrictions due to COVID-19, and testing for COVID-19 and sexually transmitted diseases—on the dosage of COVID-19 vaccine received. However, whether perceived sexual stigma and depressive symptoms before the pandemic predict MoVAC-19 warrants further examination through prospective studies. Second, MoVAC-19 among LGB individuals has not been examined using a validated scale. The Motors of COVID-19 Vaccination Acceptance Scale (MoVac-COVID19S) [24], which is based on the cognitive model of empowerment [25,26], incorporates four cognitive components determining individuals’ MoVAC-19, including values (i.e., whether individuals care about vaccination), impact (i.e., whether individuals believe in the effectiveness of vaccination), knowledge (i.e., whether individuals are knowledgeable about vaccination), and autonomy (i.e., whether individuals are confident in being vaccinated when they want to be). This scale appears to be a favorable candidate for assessing individuals’ MoVAC-19. Third, no previous studies have examined the predictive effects of sexual stigma on LGB individuals’ MoVAC-19. In the Study on Sexual Stigma and Mental Health in Taiwanese LGB Individuals, conducted in 2018 and 2019, data regarding multiple aspects of sexually stigmatizing experiences—including perceived sexual stigma from family members, internalized sexual stigma, and perceived sexual orientation microaggression—were collected, as were data related to the depressive symptoms of 1000 LGB individuals living in Taiwan [27,28,29,30,31]. Data collected before the COVID-19 pandemic can be used to examine the predictive effects of sexual stigma and depressive symptoms on LGB individuals’ MoVAC-19. Fourth, studies have indicated that sexual orientation strongly moderates the relationship between the perceived likelihood of receiving a COVID-19 vaccine and depressive symptoms and discrimination [15]. However, whether demographic-, sexual-, and gender-related characteristics moderate the predictive effects of depressive symptoms and perceived sexual stigma warrants further examination through prospective studies.

The present 4-year follow-up study examined the predictive effects of demographic characteristics (i.e., sex, age, educational level, sexual orientation, and gender orientation), depressive symptoms, and three types of sexual stigma before the COVID-19 pandemic on levels of MoVAC-19 among a group of young adult LGB individuals. We hypothesized that more severe depressive symptoms before the pandemic would predict higher MoVAC-19, whereas more severe sexual stigma before the pandemic would predict lower MoVAC-19. We also hypothesized that demographic characteristics would moderate the predictive effects of depressive symptoms and sexual stigma on MoVAC-19.

## 2. Methods

### 2.1. Participants and Procedure

The Study on Sexual Stigma and Mental Health in Taiwanese LGB Individuals, approved by the Institutional Review Board of Kaohsiung Medical University Hospital (KMUHIRB-F(II)-20180018), was a questionnaire survey study, where the questionnaire was developed based on the criteria proposed by Preston [32]. Both the criteria and methodology used for recruiting participants at baseline have been described in previous studies [27,28,29,30,31]. In brief, a cohort of 1000 individuals (500 men and 500 women) at baseline were recruited through online advertisements on social media platforms—including Facebook, Twitter, and LINE—and a bulletin board system from August 2018 to June 2019. The inclusion criteria were identifying as an LGB individual, being 20 to 30 years of age, and living in Taiwan. Early adulthood is a phase of life in which individuals pursue independence and explore life goals [33]; sexual stigma may damage the development of self-identity in early adult LGB individuals. Therefore, this study focused on the LGB individuals aged between 20 and 30 years. The exclusion criterion was having any form of impaired cognition that might have interfered with the ability to complete a questionnaire. Four years later, the same 1000 individuals were contacted by text message and invited to participate in a follow-up study. Those who responded to this message and agreed to participate received a blank consent form, a research questionnaire, and instructions for how to complete the questionnaire; they were also allowed to contact the research assistant for help if they had any problem understanding the questionnaire. Participants completed the study questionnaire individually in the study rooms and were assured that their responses would remain confidential. Only the researchers of this study could approach the data. A total of three text messages were sent to the potential participants to invite them to participate in the follow-up study, with a 1-month interval between each pair of messages. Those who responded to none of these messages were considered to have been lost to follow-up. This study was approved by the Institutional Review Board of Kaohsiung Medical University Hospital (approval number: KMUHIRB-F(I)-20210219)).

### 2.2. Measures

#### 2.2.1. Predicting Variables at Baseline

Depressive symptoms and three types of sexual stigma were measured at baseline.

##### 20-Item Mandarin Chinese Version of the Center for Epidemiologic Studies Depression Scale (MC-CES-D-20)

The present study used the MC-CES-D-20 [34,35] to assess the severity of depressive symptoms in the month preceding this study. Participants rated each item on a 4-point Likert scale with endpoints ranging from 1 (rarely or none of the time) to 4 (most or all of the time). A higher total score indicated more severe depression. The Cronbach’s α coefficient for this scale was 0.91 in this study.

##### Homosexuality-Related Stigma Scale (HRSS)

The 12-item HRSS was used to measure the participants’ levels of perceived sexual stigma from family members [36]. Each item was rated on a 4-point Likert scale with endpoints ranging from 1 (strongly disagree) to 4 (strongly agree). Scores for individual items were summed to obtain an overall HRSS score. A higher score indicated that the participant perceived a greater level of sexual stigma from family members [36]. The Cronbach’s α coefficient for this scale was 0.93 in this study.

##### Traditional Chinese Version of the Measure of Internalized Sexual Stigma for Lesbians and Gay Men (TC-MISS-LG)

The 17-item TC-MISS-LG was used to assess each participant’s sexuality, identity, and level of social discomfort [37]. Participants rated each item on a 5-point Likert scale with endpoints ranging from 1 (strongly disagree) to 5 (strongly agree). A higher score indicated a greater degree of internalized sexual stigma. Psychometric evidence supports the reliability and validity of the TC-MISS-LG for the Taiwanese population [30]. The Cronbach’s α coefficient for this scale was 0.76 in this study.

##### Traditional Chinese Version of the Sexual Orientation Microaggression Inventory (TC-SOMI)

The 19-item TC-SOMI [31,38] was used to assess LGB participants’ experiences of microaggression, including anti-LGB attitudes, denial of the existence and values of homosexuality, and public disapproval over the past 6 months. Psychometric evaluation revealed a bifactor structure for the SOMI, with a general factor distinct from the other four trait factors [38]. Participants rated each item on a 5-point Likert scale with endpoints ranging from 1 (not at all) to 5 (almost every day). A higher score indicated a greater level of microaggression. This version of the SOMI has acceptable internal consistency and concurrent validity [31]. The Cronbach’s α coefficient for this scale was 0.90 in the present study.

##### Demographic-, Sexual-, and Sex-Related Characteristics

We collected data regarding each participant’s sex, age, educational level (high school or lower level of education vs. college or higher level of education), sexual orientation (lesbian or gay vs. bisexual), and gender orientation (transgender or not).

#### 2.2.2. Outcome Variable

##### MoVac-COVID19S

The 9-item version of the MoVac-COVID19S [24] was used to assess the participants’ MoVAC-19. Participants rated each item on a 7-point scale with endpoints ranging from 1 to 7. A higher total score indicated a greater level of MoVAC-19. The MoVac-COVID19S has acceptable psychometric validity for assessing MoVAC-19 in multiple populations [39].

### 2.3. Data Analysis

We conducted statistical analyses using IBM SPSS Statistics version 24.0 (International Business Machines Corporation, Armonk, NY, USA). Participants’ demographic characteristics (i.e., sex, age, educational level, sexual orientation, and gender orientation), depressive symptoms, three types of sexual stigma, and MoVAC-19 were summarized and analyzed using descriptive statistics. The distributions of continuous variables were tested for skewness and kurtosis to determine their level of departure from a normal distribution; however, the results (i.e., absolute values of <3 for skewness and <10 for kurtosis) did not reveal any severe deviation [40]. Multivariate linear regression analysis was conducted to examine the associations of depressive symptoms, perceived familial sexual stigma, internalized sexual stigma, and perceived sexual orientation microaggression at baseline with MoVAC-19 at follow-up. As a result of significant collinearity, three types of sexual stigma were separately entered into linear regression analysis models for examination of their individual predictive effects on MoVAC-19 after the effects of demographics and depressive symptoms had been adjusted for. If demographics and depressive symptoms were significantly associated with MoVAC-19, the interactions of demographics and depressive symptoms with sexual stigma were further entered into linear regression analysis models to examine their moderating effects on the associations between sexual stigma and motivation to get vaccinated [41]. As a result of multiple comparisons, a *p* value lower than 0.0125 (0.05/4) was considered statistically significant.

## 3. Results

A total of 673 (67.3%) participants responded to the invitation and agreed to participate in the follow-up study, 167 (16.7%) responded to the invitation but refused to participate in the follow-up study, and 160 (16.0) did not respond to the invitation. No differences in sex (χ^2^ = 0.005, *p* = 0.946), age (*t* = 1.890, *p* = 0.059), or sexual orientation (χ^2^ = 2.087, *p* = 0.149) were observed between those who completed the follow-up survey and those who did not. Those who did not complete the follow-up survey were more likely to have an educational level of high school or lower (χ^2^ = 15.767, *p* < 0.001). All participants who self-identified as being transgender received the follow-up assessment.

Table 1 presents the participants’ demographics, depressive symptoms, sexual stigma, and MoVAC-19. The participants (*N* = 673) were nearly evenly distributed between two groups: men (50.1%) and women (49.9%). Their mean age was 24.8 years (standard deviation (*SD*) = 2.9 years) at baseline, and most of them had a college degree or higher (*n* = 618, 91.8%). More than half of them (*n* = 373, 55.4%) were gay or lesbian, and 19 of them (2.8%) identified as transgender. The mean MC-CES-D-20 score was 18.9 (*SD* = 11.3), the mean HRSS score was 26.8 (*SD* = 6.3), the mean TC-MISS-LG score was 35.6 (*SD* = 11.5), the mean SOMI score was 42.3 (*SD* = 11.3), and the mean MoVac-COVID19S score was 49.0 (*SD* = 9.7).

Table 2 presents the results examining the associations of demographics, depressive symptoms, and sexual stigma with MoVAC-19. According to the results of Model I, men had greater MoVAC-19 than did women. Older age was positively associated with greater MoVAC-19. Educational level, sexual orientation, gender orientation, and depressive symptoms were not significantly associated with MoVAC-19. According to the results of Model II, perceived familial sexual stigma was not significantly associated with MoVAC-19. According to the results of Model III, internalized sexual stigma was negatively associated with MoVAC-19. According to the results of Model IV, perceived sexual orientation microaggression was positively associated with MoVAC-19.

As shown in Table 3, linear regression analysis was conducted to examine the interactions between demographic characteristics (i.e., sex and age) and sexual stigma (i.e., internalized sexual stigma and perceived sexual orientation microaggression). The results indicated that none of these interactions were significantly associated with MoVAC-19, indicating that sex and age did not moderate the associations of internalized sexual stigma and sexual orientation microaggression with MoVAC-19.

## 4. Discussion

Overall, our results indicated that gay or lesbian orientation, older age, increased perceived sexual orientation microaggression, and decreased internalized sexual stigma at baseline significantly predicted increased MoVAC-19 in LGB individuals after 4 years. However, educational level, sexual orientation, gender orientation, depressive symptoms, and perceived sexual stigma from family members did not significantly predict MoVAC-19. In addition, demographic-, sexual-, and gender-related characteristics did not moderate the predictive effects. To our knowledge, this is the first prospective study to examine the predictive effects of multiple aspects of sexual stigma and depressive symptoms before the COVID-19 pandemic on LGB individuals’ MoVAC-19. Our results provide insights into LGB individuals’ MoVAC-19.

Internalized sexual stigma is a process in which LGB individuals endorse public stereotypes pertaining to their sexual orientation [42,43]. LGB individuals experience several types of sexual stigma. They may also develop internalized sexual stigma toward their sexual orientation [44]. In the present study, we discovered that greater levels of internalized sexual stigma before the COVID-19 pandemic significantly predicted lower MoVAC-19. We identified several mechanisms that may account for such a prediction. First, according to the TPB and HBM, both attitudes and subjective norms related to COVID-19 vaccination influence individuals’ MoVAC-19 [9,10]. LGB individuals experiencing internalized sexual stigma may anticipate social rejection because of their sexual minority status [1,2] and self-limited scope of social relationships [45]. In addition, they may face difficulties in learning peers’ attitudes toward vaccines against COVID-19 and norms of vaccination against COVID-19, and thus their vaccination motivation may decrease. Second, according to the HBM, barriers to vaccination negatively predict the intention to get vaccinated [10]. Internalized sexual stigma may decrease LGB individuals’ intention to access medical services [46]. Hence, such individuals may face great difficulties in visiting medical units for vaccination. Third, according to the TPB, a reduced perceived behavioral control of vaccination may reduce the motivation to get vaccinated [9]. Internalized stigma affects individuals’ self-esteem [47]. LGB individuals experiencing internalized sexual stigma may lack confidence in making vaccination decisions and controlling their vaccination behaviors. Overall, our findings highlight the importance of developing strategies aimed at preventing internalized sexual stigma in LGB individuals. Further efforts to modify outdated attitudes related to LGB sexual orientation in family, school, and the workplace are warranted. Public health intervention programs addressing attitudes to LGB sexual orientation and promoting changes in attitude toward LGB individuals may contribute to diverse affirmative cultural scripts regarding the lives of LGB individuals [48] and prevent the formation of internalized sexual stigma.

Sexual orientation microaggression is a subtle form of prejudice against LGB individuals [49,50]. Among the most common forms of sexual orientation microaggression that LGB individuals encounter are discriminatory behaviors and subtle slights associated with sexual minorities and the nullification of the stigmatized experiences of LGB individuals [38,49,50]. Perceived sexual orientation microaggression is significantly associated with low self-esteem [51,52] and a nonresponse to psychotherapy among LGB individuals [53]. In this study, we hypothesized that greater perceived sexual orientation microaggression predicts lower MoVAC-19. However, our findings contradicted our hypothesis. Sexual orientation microaggression is defined as brief and commonplace, daily, verbal, behavioral, or environmental indignities that communicate hostile, derogatory, or negative slights or insults [54]. Therefore, the capability of detecting, comparing, and identifying these indignities contributes to the awareness of sexual orientation microaggression. This capability may also contribute to LGB individuals’ awareness of the social norms of vaccination against COVID-19 and their motivation to get vaccinated. However, the proposed explanation for the prediction of perceived sexual orientation microaggression in relation to vaccination motivation warrants further study.

In this study, we did not identify a significant prediction of perceived sexual stigma from family members for MoVAC-19. Although other studies have indicated that perceived sexual stigma from family members contributes to self-identity disturbance and internalized sexual stigma in LGB individuals, adult LGB individuals may evaluate the necessity of vaccination against COVID-19 on the basis of multiple sources of information beyond the influence of familial sexual stigma. This study discovered that depressive symptoms at baseline did not significantly predict MoVAC-19 4 years later. However, this finding is not consistent with those of a previous cross-sectional study [15]. The present study assessed the participants’ self-reported depressive symptoms within the preceding month at baseline. Over the 4 years of this study, the participants’ moods may have fluctuated, resulting in a reduced association with vaccination motivation. Therefore, further research should examine the prediction of depressive disorders and unremitted depressive symptoms for vaccination motivation.

In line with the findings of a previous study [21], we discovered that compared with female sex, male sex was associated with greater MoVAC-19. In addition, older age was positively associated with greater MoVAC-19. These results indicate that similar to the general population, intervention programs aimed at enhancing MoVAC-19 should be tailored to the needs of LGB individuals, regardless of their sex or age.

The following issues regarding MoVAC-19 in LGB individuals require further study. First, how prepandemic sexual stigma influence MoVAC-19 in LGB individuals warrants study. Sexual stigma may increase LGB individuals’ worry about the stigmatization treatment in medical units and reduce their MoVAC-19. Sexual stigma may also compromise LGB individuals’ efficacy to judge the effects of vaccination and to receive vaccination. However, these possible pathways warrant study. Second, congruent with the result of a previous study that bisexual individuals had lower MoVAC-19 compared with gay and lesbian individuals [14], research has found that bisexual individuals experience different psychosocial stress compared with gay and lesbian individuals [55]. The mechanisms accounting for the effect of sexual orientation on MoVAC-19 warrant further study. Third, further study needs to examine how to enhance the MoVAC-19 of LGB individuals.

### Strengths and Limitations

The first strength of this study was the prospective study design that examined the temporal relationships between predictors and the outcome variable. This study was also the first one to examine the predictive effects, prepandemic, of multiple types of sexual stigma on MoVAC-19 in LGB individuals. However, several limitations of this study warranted discussion. First, because we collected data from a single source, our results may have been subject to shared-method variance and social desirability bias. Second, our participants were interested in the follow-up survey. Nearly one third (32.7%) of the participants at baseline did not complete the follow-up survey; those who did not complete the follow-up survey were more likely to have an educational level of high school or lower. Thus, our results may not be generalizable to all LGB individuals. Third, several factors were not evaluated at baseline, including patient history of vaccination against influenza, HIV status, and chronic illnesses; and the predictive effects of these factors on MoVAC-19 remain undetermined. For example, although the WHO listed people who have severe chronic illnesses and are immunocompromised as the high-priority group for COVID-19 vaccination [20], people with chronic illnesses or living with HIV faced the challenges of economic vulnerability, decreased social support, and interrupted treatment during the COVID-19 pandemic [56]; they may experience increased psychological distress and thus reduced motivation to get vaccinated against COVID-19. Moreover, social stigma and discrimination may also contribute to vaccine hesitancy among people living with HIV [21].

## 5. Conclusions

In this study, internalized sexual stigma, gay or lesbian orientation, and older age predicted LGB individuals’ MoVAC-19; these results were congruent with our hypothesis. However, this study found that educational level, sexual orientation, gender orientation, depressive symptoms, and perceived sexual stigma from family members did not significantly predict MoVAC-19; these results were incongruent with our hypothesis. Thus, further efforts to modify the public’s discriminative attitude toward LGB individuals are warranted. Furthermore, family education and cultural change aimed at mitigating public stigma against LGB individuals are also required to reduce internalized sexual stigma toward LGB individuals. In addition, health-care providers are recommended to develop sex- and age-specific programs aimed at improving the motivation of LGB individuals to get vaccinated against COVID-19 and to provide nondiscriminative health-care services regardless of recipients’ gender or sexual orientation.

## Figures and Tables

**Table 1:** Participants’ demographics, sexual stigma, and motivation to get vaccinated against COVID-19 (N = 673).

Variable	n (%)	Mean (*SD*)	Range
Gender			
Women	336 (49.9)		
Men	337 (50.1)		
Age at baseline (year)		24.8 (2.9)	20–30
Education level			
High school or below	55 (8.2)		
College or above	618 (91.8)		
Sexual orientation			
Bisexual	300 (44.6)		
Gay or lesbian	373 (55.4)		
Transgender	19 (2.8)		
Depression on the MC-CES-D-20		18.9 (11.3)	0–57
Perceived familial sexual stigma on the HRSS		26.8 (6.3)	10–40
Internalized sexual stigma on the MISS		35.6 (11.5)	17–76
Microaggression on the SOMI		42.3 (11.3)	19–78
Motivation to get vaccinated on the MoVac-COVID19S		49.0 (9.7)	9–63

MC-CES-D-20: 20-item Mandarin Chinese version of the Center for Epidemiologic Studies Depression Scale; HRSS: Homosexuality-Related Stigma Scale; MISS: Measure of Internalized Sexual Stigma for Lesbians and Gay Men; MoVac-COVID19S: Motors of COVID-19 Vaccination Acceptance Scale; SOMI: Sexual Orientation Microaggression Inventory.

**Table 2:** Associations of demographic characteristics, depression, and sexual stigma with motivation to get vaccinated against COVID-19: multiple linear regression analysis.

	Motivation to Get Vaccinated against COVID-19							
	Model I		Model II		Model III		Model IV	
Variable	B (SE)	*p*	B (SE)	*p*	B (SE)	*p*	B (SE)	*p*
Gender ^a^	3.401 (0.771)	<0.001	3.311 (0.773)	<0.001	4.629 (0.870)	<0.001	3.127 (0.768)	<0.001
Age	0.389 (0.131)	0.003	0.381 (0.131)	0.004	0.397 (0.130)	0.002	0.399 (0.130)	0.002
Education level ^b^	1.707 (1.390)	0.220	1.676 (1.389)	0.228	1.961 (1.385)	0.157	1.981 (1.379)	0.151
Sexual orientation ^c^	0.129 (0.785)	0.869	0.135 (0.784)	0.864	−0.380 (0.799)	0.634	0.077 (0.778)	0.921
Transgender	−0.181 (2.220)	0.935	−0.084 (2.219)	0.970	−0.325 (2.208)	0.883	0.225 (2.201)	0.919
Depression	0.036 (0.033)	0.268	0.026 (0.034)	0.445	0.063 (0.034)	0.064	−0.001 (0.034)	0.976
Familial sexual stigma	–	–	0.093 (0.060)	0.120	–	–	–	–
Internalized sexual stigma	–	–	–	–	−0.111 (0.037)	0.003	–	–
Sexual orientation microaggression	–	–	–	–	–	–	0.127 (0.034)	<0.001
F	6.322		5.778		6.756		7.535	
*p*-value	<0.001		<0.001		<0.001		<0.001	
Adjusted R^2^	0.045		0.047		0.057		0.064	

B: regression coefficient; SE: standard error. ^a^ Female sex as a reference. ^b^ High school or lower as a reference. ^c^ Bisexual as a reference.

**Table 3:** Moderating effects of gender and age on the associations of internalized sexual stigma and microaggression with motivation to get vaccinated against COVID-19: multiple linear regression analysis.

	Motivation to Get Vaccinated against COVID-19			
	Model IV		Model V	
Variable	B (SE)	*p*	B (SE)	*p*
Gender ^a^	4.297 (2.668)	0.108	2.603 (2.862)	0.363
Age	0.825 (0.420)	0.050	0.377 (0.496)	0.448
Education level ^b^	2.066 (1.395)	0.139	1.963 (1.390)	0.158
Sexual orientation ^c^	−0.407 (0.800)	0.612	0.075 (0.780)	0.923
Transgender	−0.333 (2.214)	0.881	0.230 (2.205)	0.917
Depression	0.064 (0.034)	0.059	−0.001 (0.034)	0.985
Internalized sexual stigma	0.185 (0.282)	0.513	–	–
Sexual orientation microaggression	–	–	0.108 (0.276)	0.696
Gender x Internalized sexual stigma	0.009 (0.073)	0.901	–	–
Age x Internalized sexual stigma	−0.012 (0.011)	0.284	–	–
Gender x Sexual orientation microaggression	–	–	0.012 (0.066)	0.850
Age x Sexual orientation microaggression	–	–	0.001 (0.011)	0.963

B: regression coefficient; SE: standard error. ^a^ Female sex as a reference. ^b^ High school or lower as a reference. ^c^ Bisexual as a reference.

## Data Availability

The data will be available upon reasonable request to the corresponding authors.
